# Overexpression of *SNTG2, TRAF3IP2,* and *ITGA6* transcripts is associated with osteoporotic vertebral fracture in elderly women from community

**DOI:** 10.1002/mgg3.1391

**Published:** 2020-06-30

**Authors:** Levi H. Jales Neto, Zofia Wicik, Georgea H. F. Torres, Liliam Takayama, Valéria F. Caparbo, Neuza H. M. Lopes, Alexandre C. Pereira, Rosa M. R. Pereira

**Affiliations:** ^1^ Bone Metabolism Laboratory Rheumatology Division Faculdade de Medicina FMUSP Universidade de Sao Paulo Sao Paulo SP Brazil; ^2^ Faculdade de Medicina Instituto do Coracao (InCor) Hospital das Clinicas HCFMUSP Universidade de Sao Paulo Sao Paulo SP Brazil; ^3^ Laboratory of Genetics and Molecular Cardiology Faculdade de Medicina Instituto do Coracao (InCor) Hospital das Clinicas HCFMUSP Universidade de Sao Paulo Sao Paulo SP Brazil

**Keywords:** microarray study, osteoporosis, RNA expression, vertebral fracture

## Abstract

**Background:**

Vertebral fractures (VFs) are the most common clinical manifestation of osteoporosis associated with high morbimortality. A personal/familiar history of fractures increases the risk of fractures. The purpose of this study is to identify possible molecular markers associated with osteoporotic VFs in elderly women from community.

**Methods:**

Transcriptomic analysis using Affymetrix HTA2 microarray was performed using whole blood samples of 240 subjects from a population‐based survey (Sao Paulo Ageing & Health [SPAH] study). Only elderly women with osteoporosis diagnosis by densitometry were analyzed, and divided in two groups: VF: women with osteoporosis and VFs versus no vertebral fracture (NVF): women with osteoporosis and NVFs. They were matched for age, chronic disease, medication use, and bone mineral density (BMD). The logistic regression model adjusted for age was applied for transcriptome data analysis. SYBR green‐based quantitative polymerase chain reaction (qPCR) was used to validate the most significant expression changes obtained in the microarray experiment.

**Results:**

Microarray analysis identified 142 differentially expressed genes (DEGs, *p* < .01), 57 upregulated and 85 downregulated, compared VF versus NVF groups. The DEG with the greatest expression difference was the Gamma2‐Syntrophin (*SNTG2*) (*β* = 31.88, *p* = .005). Validation by qPCR confirmed increased expression in VF group of Syntrophin (*SNTG2*, fold change = 2.79, *p* = .009), TRAF3 Interacting Protein2 (*TRAF3IP2*, fold change = 2.79, *p* = .020), and Integrin Subunit Alpha 6 (*ITGA6*, fold change = 2.86, *p* = .038).

**Conclusion:**

Our data identified and validated the association of *SNTG2* (608715), *TRAF3IP2* (607043), and *ITGA6* (147556) with osteoporotic VF in elderly women, independently of BMD. These results suggest that these transcripts have potential clinical significance and may help to explain the molecular mechanisms and biological functions of vertebral fracture.

## Graphical abstract

Our data identified and validated the association of SNTG2, TRAF3IP2, and ITGA6 with osteoporotic vertebral fracture (VF) in elderly women, independently of bone mineral density. These results suggest that these transcripts have potential clinical significance and may help to explain the molecular mechanisms and biological functions of VF.

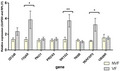

## 1 INTRODUCTION

Osteoporosis is a bone disorder characterized by low bone density and microarchitecture deterioration, which may result in fragility and fracture risk, occurring most commonly in postmenopausal women (Brunton et al., 2005).

Vertebral fractures (VFs) are the most common osteoporotic fractures. The prevalence in the elderly varies between 10% and 30%, according to age and gender (Felsenberg et al., 2002; Samelson et al., 2006; Van der Klift, De Laet, McCloskey, Hofman, & Pols, 2002). Fractures are associated with high morbidity, (Gold, 1996; Oleksik, Ewing, Shen, van Schoor, & Lips, 2005; Ross, 1997) including acute and chronic pain, loss of independence, height loss, kyphosis, depression, and high mortality (Bliuc, Nguyen, Milch, Nguyen, & Eisman, 2009; Ensrud et al., 2000). Additionally, VFs are expected to be an essential health problem for aging societies (Burge et al., 2007). It is well known that fracture at the spine is associated with the incidence of new osteoporotic fractures in other regions (Domiciano et al., 2014; Melton, Atkinson, Cooper, O'Fallon, & Riggs, 1999).

The Framingham Heart Study showed that VF risk, volumetric bone mineral density (BMD), and geometry at the lumbar spine are complex genetic traits related to gene‐environment interactions (Liu et al., 2011). Family history of a VF represents an independent risk factor for incident fractures, (Kanis et al., 2004) showing the significance of heredities in the pathogenesis of bone metabolism deterioration. A genome‐wide association study (GWAS) identified 56 BMD loci and reveals 14 loci associated with the risk of all fractures (Estrada et al., 2012).

To date, there are no studies in the literature analyzing the processes associated with VFs on the transcriptomic level. Based on that, the aim of this work was to find differentially expressed genes (DEGs) and pathways associated with osteoporotic VFs.

## 2 METHODS

### 2.1 Subjects

This study was conducted from June 2015 to July 2016 and subjects were selected from a previous epidemiologic project, which included a population‐based survey (São Paulo Ageing & Health [SPAH] study). Two hundred and forty individuals were evaluated and RNA samples were extracted. Of them, 92 men were excluded to avoid comparison with different sexes, 20 women were excluded due to low RNA concentration (<100 ng/dl), 34 women were excluded due to chip hybridization failure, 4 women were excluded in normalization from the data for bioinformatics, 50 women were excluded for BMD T‐score >−2.5, and 4 women were excluded for presenting only grade 1 fracture. Therefore, 36 elderly women (≥65‐year age) with osteoporosis diagnosis by densitometry were analyzed. They were divided into two age‐matching groups: (a) vertebral fracture (VF, *n* = 24): osteoporosis with VF and (b) no vertebral fracture (NVF, *n* = 12): osteoporosis with NVF.

All subjects responded to a standardized questionnaire regarding lifestyle and health, including history of hip fracture, fragility and previous fractures during last year, physical activity, alcohol intake, falls in the last 12 months, smoking status, personal history of hypertension, diabetes mellitus cardiovascular events (myocardial infarction and cerebrovascular disease), dietary calcium intake, menopause age, and all were apparently healthy. No individual had malabsorption or chronic diarrhea or hepatic disease or had severe chronic diseases or cancer or using glucocorticoid. BMD and VFs were assessed by dual‐energy X‐ray absorptiometry (DXA). Race was defined based on the self‐reported race of second‐generation ancestors, an approach previously used for the Brazilian population. Individuals with four grandparents reported as White were classified as White, while those individuals with Black and White ancestors (mixed race) were classified as non‐White. Individuals with Asian ancestors were also classified as non‐White. When racial information regarding the grandparents was not available, an individual's race was determined by the race of his or her parents (Lopes et al., 2011).

The Local Ethics in Research Committee of the São Paulo University School of Medicine approved this study, and all participants gave written informed consent.

## 3 MATERIAL AND METHODS

### 3.1 Blood collection, RNA extraction, and microarray analysis

Twenty milliliter of peripheral whole blood samples was collected under fasting conditions (between 8:00 a.m. and 10:00 a.m.) and stored at −80°C until analysis. These samples were used to perform biochemical tests of bone metabolism (calcium, phosphorus, alkaline phosphatase, 25OHD, iPTH, CTX, P1NP) and RNA extraction. About 10 ml was collected in PAXgene Blood RNA tubes, and total RNA was extracted, isolated, and purified with RNeasy columns (Qiagen) and treated with RNase‐Free DNase Set (Qiagen). Bioanalyzer 2100 System (Agilent) to analyze ribosomal bands 28S and 18S was used to confirm purified RNA. Samples with a minimum of 100 ng of RNA underwent in vitro transcription with the Ambion WT Expression kit (Life Technologies) in Affymetrix GeneChip*^®^* WT Terminal Labeling and Control kit. The RNA quality was evaluated with the NanoDrop spectrophotometer (ND‐1000, NanoDrop, Thermo Scientific). Labeled samples were hybridized to the Affymetrix GeneChip*^®^* Human Transcriptome Array HTA 2.0. Array hybridization, washing, staining, and scanning were done as per subsequent Affymetrix instructions. 67,528 transcribed genes covering 44,699 coding genes and 22,829 noncoding regions were analyzed. After quality control and assessment of the expression signals, CEL files have been subjected to further analysis using Affymetrix GCOS*^®^*.

### 3.2 Evaluation of the serum biochemical bone parameters (calcium, phosphorus, and alkaline phosphatase)

The concentrations of serum calcium, phosphorus, and alkaline phosphatase were measured using standard automated laboratory methods. Serum concentrations of 25‐hydroxyvitamin D (25OHD) and iPTH were determined using a radioimmunoassay technique (DiaSorin) and an immunoradiometric assay (ELISA‐PTH; CIS Bio International), respectively. C‐terminal telopeptide of type I collagen (CTX) and N‐terminal propeptide of type I procollagen (P1NP) serum concentrations were measured by automated Electrochemiluminescence (E411, Roche Diagnostics). The serum CTX limit of detection was 10 ng/ml and intra‐assay and inter‐assay coefficients variation (CVs) were 2.5% and 3.4%, respectively. Serum P1NP intra‐assay and inter‐assay CVs were 2.2 and 1.8%, respectively, with a limit of detection of 5% µg/L.

### 3.3 BMD and semi‐quantitative measurements for VF

Bone mineral density was determined by DXA, using a Hologic QDR 4500A densitometry equipment (Hologic Inc., Discovery model) at the lumbar spine, femoral neck, and total hip. The precision errors for BMD measurements were measured based on the standard protocols from The International Society for Clinical Densitometry (ISCD) (Bennett et al., 2006). The prevalent VF was assessed from T4 to L4, a Vertebral Fracture Assessment (VFA) scan of the thoracolumbar spine performed using the same DXA device, with subjects in the supine position. All DXA measurements were performed by the same experienced technologist (LT). On that same week, standard lateral thoracic and lumbar spine radiographs were taken using a 40‐inch tube‐to‐film distance centered at T7 and L2. All VFA and radiographic images were independently evaluated by two experienced rheumatologists (GHM and LHJN). For the identification of VFs, in both methods, the readers assessed each T4–L4 image to decide whether a fracture was present. A consensus was agreed between the readers for any difference in interpretation. Interobserver agreement was 96% and the Kappa coefficient was 0.83. Nonvisible vertebrae were excluded. Only adequately visualized vertebrae were analyzed for deformity using the Genant semi‐quantitative (SQ) method (Genant, Wu, Vankuijk, & Nevitt, 1993). Each identified fractured vertebra was classified by grade based on the Genant SQ scale, where mild (grade 1) is a reduction of 20%–25% of anterior, middle, and/or posterior height relative to the adjacent vertebral bodies; moderate (grade 2) is a reduction of 26%–40% in any height; and severe (grade 3) is a reduction of 40% in any height. For this study, grade I fractures were excluded; therefore, subjects with grade II or grade III VFs were included in the VF group and subjects without any vertebral deformity were included in the NVF group.

### 3.4 Microarray data preprocessing and analysis

The raw data were preprocessed in R with affy and multtest packages from Bioconductor (Parmigiani, Garrett, Irizarry, & Zeger, 2003). Quality control of individual arrays included various advanced quality metrics, diagnostic plots, and pseudo‐images to certify that only excellent quality arrays were used prior to downstream statistical analysis. The robust multiarray average (RMA) algorithm was used for background correction and quantile normalization, and median polish summarization was made using the software expression console. The raw intensity of each probe was processed by R program (version v2.12.1; https://www.r‐project.org). Probes that passed the criteria were normalized by 75% median scaling normalization method. Normalized spot intensities were transformed to gene expression log 2 ratios between the control and fracture groups. The probes with log 2 ratio ≥1 or log 2 ratio ≤−1 and *p* < .01 were defined as differential genes for additional pathway enrichment analysis. In this section, we present the results of the adjustment of the logistic model taking into account the binary variables, osteoporosis and VF (Irizarry et al., 2003).

Statistical analysis of the data was performed in R by using a logistic regression model taking into account age adjustment. In this approach, downregulation reflects an association with no risk of VF, whereas upregulation reflects an association with VF presence. The probes with *β* ≥1 or *β* ≤−1 and *p* ≤ .01 were defined as DEGs and after their identification, additional pathway enrichment analysis was performed.

### 3.5 Gene ontology and biological pathways enrichment analysis

In further steps of the analysis, DEGs (gene symbols with *β* ratios) were uploaded into the Ingenuity Pathway Analysis (IPA) software (Ingenuity Systems) to identify relevant differential expression pathways. Gene ontology (GO) analysis and pathway enrichment analysis are commonly used for functional studies of large‐scale transcriptomic or genomic data (Hulsegge, Kommadath, & Smits, 2009). We performed these analyses using up‐ and downregulated DEGs at *p* ≤ .01 adjusted for multiple comparisons. DEGs were also enriched in the biological process (BP), cellular component (CC), and molecular function (MF) categories of GO database. FUMA GWA was used to search for overrepresentation of the hallmark gene sets, biological pathways. The Database Functional Mapping and Annotation of Genome‐Wide Association Studies (FUMA GWA, http://fuma.ctglab.nl) provides a set of data‐mining tools and assists in the interpretation of genome‐scale datasets by expediting the transition from data collection to biological annotation. GO terms with *p* < .05 based on a hypergeometric test were defined to be statistically significant. Basically, upregulation reflects an association with risk of developing VFs and downregulation revels decreased risk of VF in women with osteoporosis.

### 3.6 Interaction network analysis

The interaction network for DEGs was constructed using Cytoscape 3.6 software and stringApp implementing 1.4 String interaction database (Shannon et al., 2003). Interaction confidence cutoff was set as ≥0.4. Tissue expression confidence levels were obtained from stringApp. Age‐related expression changes in muscle tissue were obtained from the GTEX study (Yang et al., 2016).

### 3.7 Quantitative polymerase chain reaction assays of significantly differentially expressed mRNAs

cDNA was synthesized from 100 ng of RNA using cDNA Reverse Transcription kits (Applied Biosystems) and thermocycler PTC‐100 (MJ Research) was used for cDNA synthesis. Quantitative PCR was performed using PowerUp ™ SYBR ™ Green Master Mix (Thermo Fisher Scientific) on the StepOne Plus quantitative polymerase chain reaction (qPCR) (Applied Biosystems) cycler according to the manufacturer's instructions. All samples were tested in triplicates.

PCR primers for tested genes were designed based on their DE microarray probe exomic location. Primers were designed using Primer3, OligoCalc, OligoAnalyzer, and Primer‐BLAST (Owczarzy, 2018) online tools. Primer sequences are in Table S1. To validate the changes in gene expression, we used the Pfaffl method for relative expression quantification (Pfaffl, 2001). The geometric mean of the two most stable housekeeping genes according to microarrays (*RPL27* and *GAPDH*) was used to compare the gene expression between the groups (Hellemans, Mortier, De Paepe, Speleman, & Vandesompele, 2007). *p*‐Values were calculated using an unpaired *t* test and 95% confidence intervals were calculated in R assuming unequal variances.

## 4 RESULTS

### 4.1 Subjects’ characteristics

From the 36 women with osteoporosis, 24 presented VFs and 12 had NVFs. No difference between the two groups (VF vs. NVF) was observed regarding the lumbar spine, femoral neck, and total hip BMD, frequency of chronic disease, life habits such as smoking, alcoholism, physical activity, and the number of falls. No differences were also observed in biochemical and bone turnover markers (calcium, phosphorus, PTH, alkaline phosphatase, 25‐hydroxyvitamin D, P1NP, and CTX). Table 1 presents the participants’ characteristics.

**Table 1:** Clinical and biochemical features of older women with osteoporosis and Vertebral Fracture (VF) and osteoporosis with No Vertebral Fractures (NVFs) participating in the study

Variables	VF	NVF	*p*‐value
Sample size	(*n* = 24)	(*n* = 12)	
Age (years)	81.46 4	81.4 3.1	1
Race			.104
White	7 (29.2%)	7 (58.3%)	
No white	14 (58.3%)	5 (41.7%)	
Asian	3 (12.5%)	0 (0%)	
Height (cm)	147.4 ± 5.9	146.0 ± 3.1	.365
Weight (kg)	61.1 ± 11.8	57.2 ± 10.4	.339
BMI (kg/m2)	28.1 ± 5.2	26.8 ± 4.6	.468
Alcohol (≥3U/day), %	4 (16.7%)	2 (16.7%)	1
Smoking or ex‐smoking (%)	3 (12.5%)	1 (8.3%)	1
Physical activity			.771
Low	5 (20.8%)	3 (25%)	
Moderate	15 (62.5%)	8 (66.7%)	
High	4 (16.7%)	1 (8.3%)	
Fall (≥1/year), %	5 (20.8%)	3 (25%)	1
Osteoporosis family history (%)			.379
No	15 (62.5%)	10 (83.3%)	
Yes	3 (12.5%)	1 (8.3%)	
Unknown	6 (25%)	1 (8.3%)	
Fracture family history (%)			.192
No	16 (66.7%)	11 (91.7%)	
Yes	1 (4.2%)	0 (0%)	
Unknown	7 (29.2%)	1 (8.3%)	
Diabetes Mellitus (%)	3 (12.5%)	3 (25%)	.378
Hypertension (%)	19 (79.2%)	11 (91.7%)	.64
Heart attack (%)	3 (12.5%)	1 (8.3%)	1
Stroke (%)	2 (8.3%)	0 (0%)	.543
Dyslipidemia (%)	11 (45.8%)	7 (58.3%)	.48
Calcium supplement use (%)	10 (41.7%)	6 (50%)	.635
Vitamin D supplement use (%)	15 (62.5%)	6 (50%)	.473
Bisphosphonates use (%)	11 (45.8%)	5 (41.7%)	.813
Creatinine (mg/dl)	0.8 (0.73–1.09)	0.79 (0.62–0.89)	.47
Total calcium (mg/dl)	9.6 (9.5–9.8)	9.6 (9.3–9.98)	.853
Total phosphorus (mg/dl)	3.6 (3.2–3.78)	3.5 (3.15–3.68)	.479
Alkaline phosphatase (U/L)	81 (61.75–94.25)	68.5 (62.25–86.75)	.557
25OHD (ng/ml)	23.5 ± 6.8	19.0 ± 9.0	.101
iPTH (pg/dl)	57.3 ± 22.3	60.1 ± 14.3	.696
CTX (ng/ml)	0.27 ± 0.16	0.33 ± 0.19	.303
P1NP (ng/ml)	36.65 (17.95–48.55)	38.75 (22.9–58.83)	.505
Lumbar spine BMD (g/cm^2^)	0.77 ± 0.11	0.77 ± 0.15	.956
Lumbar spine T‐score	−2.89 ± 1.08	−2.79 ± 1.05	.804
Total hip BMD (g/cm^2^)	0.71 ± 0.11	0.70 ± 0.08	.862
Total hip T‐score	−2.13 ± 0.81	−2.02 ± 0.62	.701
Femoral neck BMD (g/cm^2^)	0.57 ± 0.10	0.59 ± 0.06	.663
Femoral neck T‐score	−2.60 ± 0.74	−2.42 ± 0.57	.477

Note

The Kolmogorov‐Smirnov test was used to evaluate the normal distribution. The values of p are for comparisons of means (Student's *t*‐test or Mann‐Whitney test) or proportions (Chi‐square test, Fisher's test, or likelihood ratio test). Values of *p* < .05 were considered significant.

Abbreviations: 25OHD, 25‐hydroxyvitamin D; BMD, bone mineral density; BMI, body mass index; iPTH, intact parathyroid hormone.

### 4.2 Identification of VFs

Vertebral fractures were detected using Vertebral Fracture Assessment, using the Genant criteria (Genant & Jergas, 2003). From 24 women, 20 presented VFs grade 2 and four women presented VF grade 3. The VFs were found in 72% participants at the thoracic level and 28% at the lumbar level, with biconcave‐shape in 49% and wedge‐shape in 51%.

### 4.3 DEG analysis

**Figure 1:**
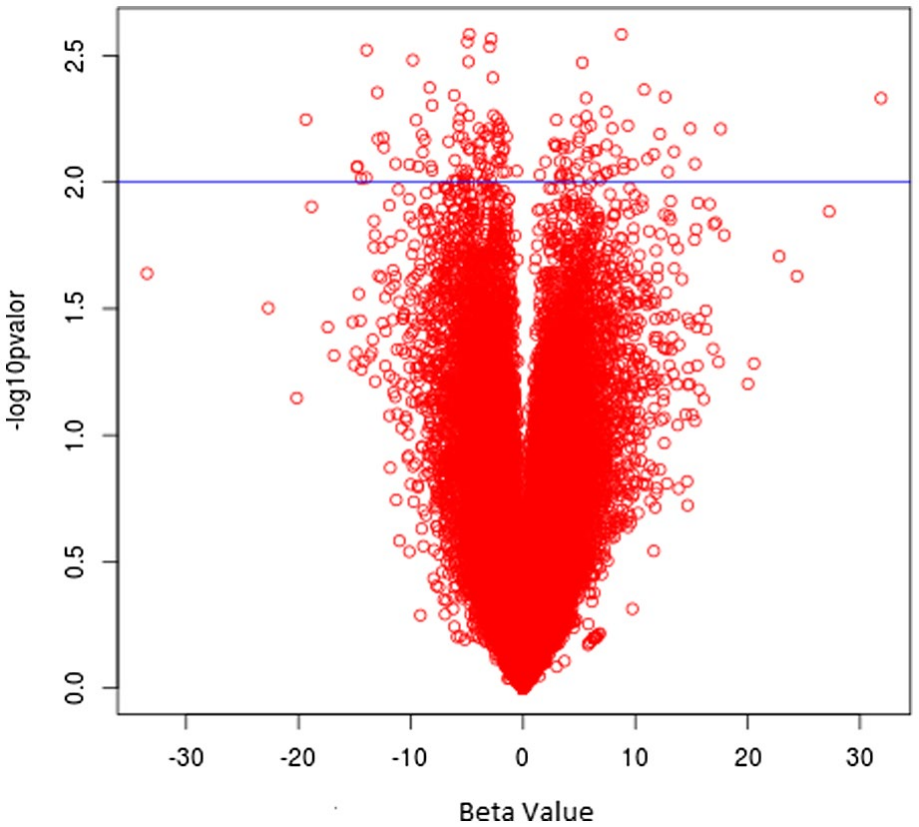
Up‐ and downregulated DEGs: Vulcan plot with all analyzed genes. Blue line indicates threshold of significant (*p* < .01) DEGs. Fifty‐seven DEGs are upregulated (right side), and 85 DEGs are downregulated (left side). DEG, differentially expressed gene

We identified 142 DEGs in the comparison between VF and NVF groups. Among them, 57 were upregulated and 85 downregulated (Figure 1). The top 10 up‐ and downregulated DEGs with the highest *β* are shown in Table 2. We found that SNTG2 expression had the highest *β* of association (*β* = +31.88, *p* = .01). As a potential protection factor, the gene Rho GTPase activating protein 8 (*β* = −19.35, *p* = .01) was found as the highest downregulated gene.

**Table 2:** The top 10 differentially expressed genes (DEGs) between older women with osteoporosis and vertebral fracture (VF) and osteoporosis with no vertebral fractures (NVFs)

Genes	Symbol	(*β*)	Regulated	*p*‐value
Syntrophin gamma 2	*SNTG2*	31.88	Up	.004
Prader‐Willi region nonprotein coding RNA 1	*PWRN1*	17.6	Up	.006
Keratin‐Associated Protein 19‐1	*KRTAP19‐1*	15.31	Up	.008
Lipocalin 1	*LCN1*	14.89	Up	.006
Ectonucleotide Pyrophosphatase/Phosphodiesterase 2	*ENPP2*	13.45	Up	.007
Cysteine Rich Tail 1	*CYSRT1*	12.91	Up	.009
CD248 Molecule	*CD248*	12.64	Up	.004
TRAF3 Interacting Protein 2	*TRAF3IP2*	12.18	Up	.006
Partner Of NOB1 Homolog	*PNO1*	11.66	Up	.007
Glycoprotein V Platelet	*GP5*	11.09	Up	.008
Rho GTPase activating protein 8	*ARHGAP8/PRR5‐ARHGAP8*	−19.35	Down	.005
MTOR‐associated protein, LST8 homolog	*MLST8*	−14.8	Down	.008
Phospholipid Transfer Protein	*PLTP*	−14.73	Down	.008
Synapse defective 1, Rho GTPase, homolog 1 (C. elegans)	*SYDE1*	−14.41	Down	.009
Glycosylphosphatidylinositol‐Specific Phospholipase D1	*GPLD1*	−13.96	Down	.009
Zinc Finger Protein 653	*ZNF653*	−12.91	Down	.003
Deleted In Primary Ciliary Dyskinesia Homolog	*DPCD*	−12.45	Down	.004
Serine/Threonine Kinase 16	*STK16*	−12.39	Down	.006
Alpha‐1,4‐N‐Acetylglucosaminyltransferase	*A4GNT*	−11.35	Down	.006
ECSIT Signaling Integrator	*ECSIT*	−9.52	Down	.007

Enrichment analysis of all DEGs showed overrepresentation of the mTOR1 signaling in pathway (*p‐*adjusted = .03) with the influence of *PNO1* and *PDK1*. Wiki pathway enrichment analysis was identified as the most significantly enriched focal adhesion pathway (*p‐*adjusted = .03) influenced by *ITGA6* and *TNXB*. Moreover, *LCN1*, *ITGA6*, and *TNXB* were also found upregulated in women with VFs compared with women with NVFs. Analysis of the canonical pathways showed enrichment in KEGG ECM receptor interaction, KEGG taste transduction, and KEGG hematopoietic cell lineage with *ITGA6, GP5, TNXB, TAS2R60,* and *TAS2R4* involved. Enrichment of GO analyze BP analysis showed regulation of phosphorus metabolism process as the pathway with the most genes involved (*p*‐adjusted = .03, with six genes in this pathway: *WNK1, ITGA6, PDK1, PLCL1, TNXB,* and *ENPP2*) and in GO MF, the binding receptor pathway (*p*‐adjusted = .04, with five genes in this pathway: *SNTG2, PLCL1, ITGA6, TNFB,* and *TRAF3IP2*) was significantly expressed in VF group. A total of 24 significant pathways (*p*‐adjusted < .05) were found, seven in BP, five in CCs, and 12 in MF. GO BP: sensory perception of taste (genes: *TAS2R60, TAS2R41,* and *LCN*), detection of chemical stimulus involved in sensory perceptions of taste (genes: *TAS2R60* and *TAS2R41*), regulation of phosphorus metabolic process (genes: *WNK1, ITGA6, PDK1, PLCL1, TNXB,* and *ENPP2*), cell matrix adhesion (genes: *ITGA6* and *TNXB*), glycerolipid metabolic process (genes: *SERINC5, TNXB,* and *ENPP2*), cell substrate adhesion (genes: *ITGA6* and *TNXB*), and organelle disassembly (genes: *PDK1* and *FAM134B*). No GO CCs: proteinaceous extracellular matrix (genes: *CD248, FBLN7, ITGA6,* and *TNXB*), extracellular matrix (genes: *CD248, FBLN7, ITGA6,* and *TNXB*), extracellular matrix component (genes: *ITGA6* and *TNXB*), cell substrate junction (genes: *RPS29, FBLN7,* and *ITGA6*), and anchoring junction (genes: *RPS29, FBLN7,* and *ITGA6*). GO MF: bitter taste receptor activity (genes: *TAS2R60* and *TAS2R41*), taste receptor activity (genes: *TAS2R60* and *TAS2R41*), phosphoric diester hydrolase activity (genes: *PLCL1* and *ENPP2*), phospholipase activity (genes: *PLCL1* and *ENPP2*), carbohydrate binding (*CD248, LGALS14,* and *ENPP2*), integrin binding (genes: *ITGA6* and *TNXB*), lipase activity (genes: *PLCL1* and *ENPP2*), heparin binding (*FBLN7* and *TNXB*), cell adhesion molecule binding (genes: *ITGA6* and *TNXB*), glycosaminoglycan binding (genes: *FBLN7* and *TNXB*), sulfur compound binding (genes: *FBLN7* and *TNXB*), and receptor binding (*SNTG2, ITGA6, PLCL1, TNXB,* and *TRAF3IP2*). The most frequent genes were *TNXB* found in 54% of the pathways and *ITGA6* in 50% (Table S2).

### 4.4 Interaction network analysis

**Figure 2:**
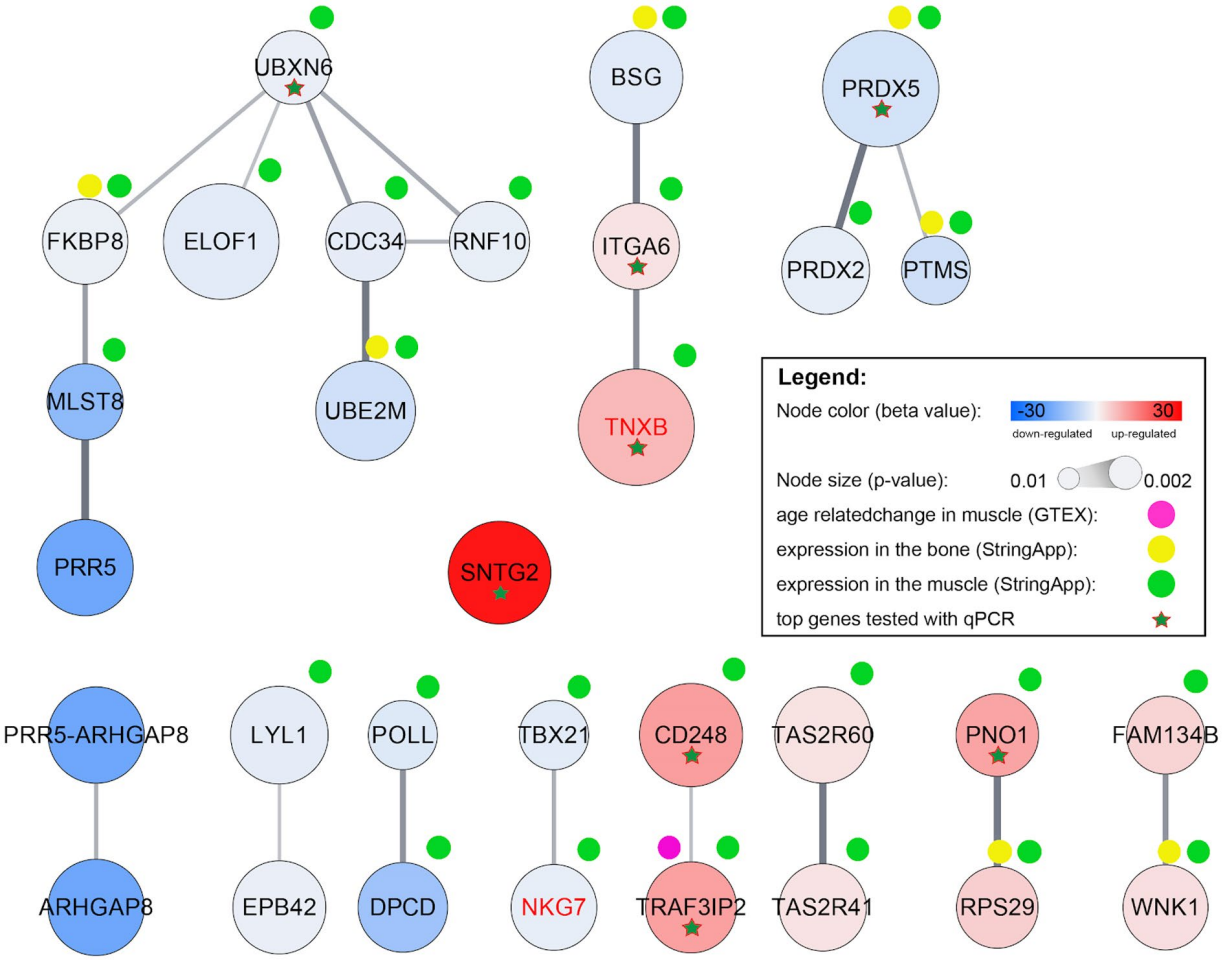
Interaction network of the genes differentially expressed (*p* < .01) between the older women with osteoporosis and vertebral fracture (VF) and osteoporosis with no vertebral fractures (NVFs). On the figure are shown genes with at least one neighbor and differentially expressed interactors selected for qPCR validation. qPCR, quantitative polymerase chain reaction

An interaction network constructed from all DEGs showed significant enrichment of interactions between the analyzed genes (PPI enrichment *p*‐value = .00121). Among interacting DEG, we observed especially significant functional enrichment (*p* FDR corrected ≤.05) within one network containing eight downregulated genes. This enrichment was observed for partially overlapped ubiquitin signaling (*UBXN6, CDC34,* and *UBE2M*), adaptive immune system (*CDC34, UBE2M,* and *PRR5*), and antigen processing (*CDC34* and *UBE2M*). Other enriched processes associated with interacting DEGs were redoxin functions (*PRDX5* and *PRDX2*) and extracellular matrix binding (*ITGA6* and *CD248*). All of the genes associated with those processes also showed expression in the muscle tissue. *PRDX5* and *UB2M* also showed expression in bone tissue according to StringApp (Figure 2).

### 4.5 Validation of differential gene expression using qPCR

**Figure 3:**
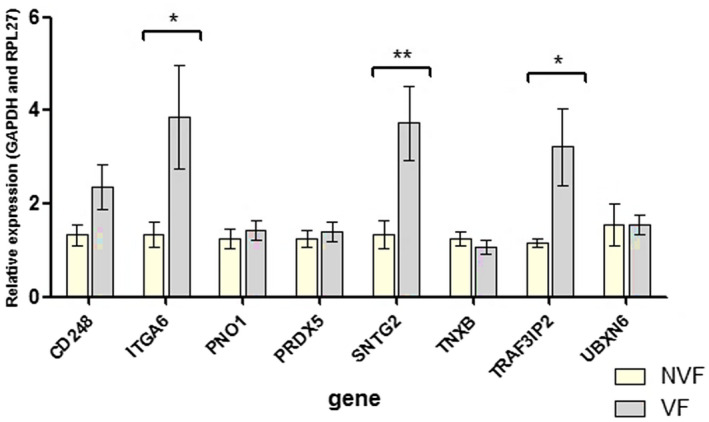
Results for the genes selected for qPCR‐RT (quantitative real‐time polymerase chain reaction). *Genes Validated. qPCR, quantitative polymerase chain reaction

For qPCR validation, we selected the eight DEGs with the highest *β* ratios which were also highlighted in enrichment and/or interaction network analysis. We analyzed six upregulated mRNAs (*SNTG2, TRAF3IP2, PNO1, CD248, TNXB,* and *ITGA6*) and two downregulated mRNA (*PRDX5* and *UBXN6*). To determine the expression levels of these genes, we performed qPCR (VF: *n* = 19, Control: *n* = 12). We chose *RPL27* and *GAPDH* as the endogenous control gene for the real‐time qPCR because its expression levels in microarrays were very consistent and stable across all the samples. Six DEGs (*SNTG2; CD248; TRAF3IP2; PNO1; ITGA6; UBXN6*) showed the direction of the regulation of gene expression consistent with microarray results. Three of them, *SNTG2, ITGA6,* and *TRAF3IP2*, showed statistically significant (*p* < .05) changes in expression in the qPCR analysis. *CD248* was almost significant at the 0.05 level (*p* = .0588). Expression changes of the two DEGs (*PRDX5* and *TNXB*) were not confirmed. The results of qPCR analysis are shown in Figure 3 and in Table 3.

**Table 3:** Summary of the results for the genes selected for qPCR‐RT (quantitative real‐time polymerase chain reaction)

Gene symbol	Top 10: Upregulated	Top 10: Downregulated	Interaction network (Cytoscape and StringApp)	Muscle expression (String database)	Bone expression (String database)	Enrichment analysis (FUMA)	Aging‐Related changes in expression in muscle tissue (GTEX)	qPCR RQ = fold change compared to the calibrator (RQ SEM)	qPCR *p*‐value
*CD248*	Yes		Yes	Yes (score 3)				1.78 (0.52)^a^	.059
*ITGA6*			Yes	Yes (score 2)		Yes		2.86 (1.14)^a^	.038^a^
*PNO1*	Yes		Yes	Yes (score 1)				1.12 (0.30)^a^	.622
*PRDX5*		Yes	Yes	Yes (score 3)				1.14 (0.27)	.537
*SNTG2*	Yes					Yes		2.79 (0.86)^a^	.009^a^
*TNXB*			Yes	Yes (score 2)		Yes		0.86 (0.21)	.414
*TRAF3IP2*	Yes		Yes	Yes (score 2)		Yes	Yes	2.79 (0.83)^a^	.020^a^
*UBXN6*		Yes	Yes	Yes (score 3)	Yes (score 1)			0.99 (0.49)^a^	.981

Abbreviation: qPCR, quantitative polymerase chain reaction.

^a^ Confirmation of the microarray analysis.

### 4.6 ROC curve qPCR‐RT

**Figure 4:**
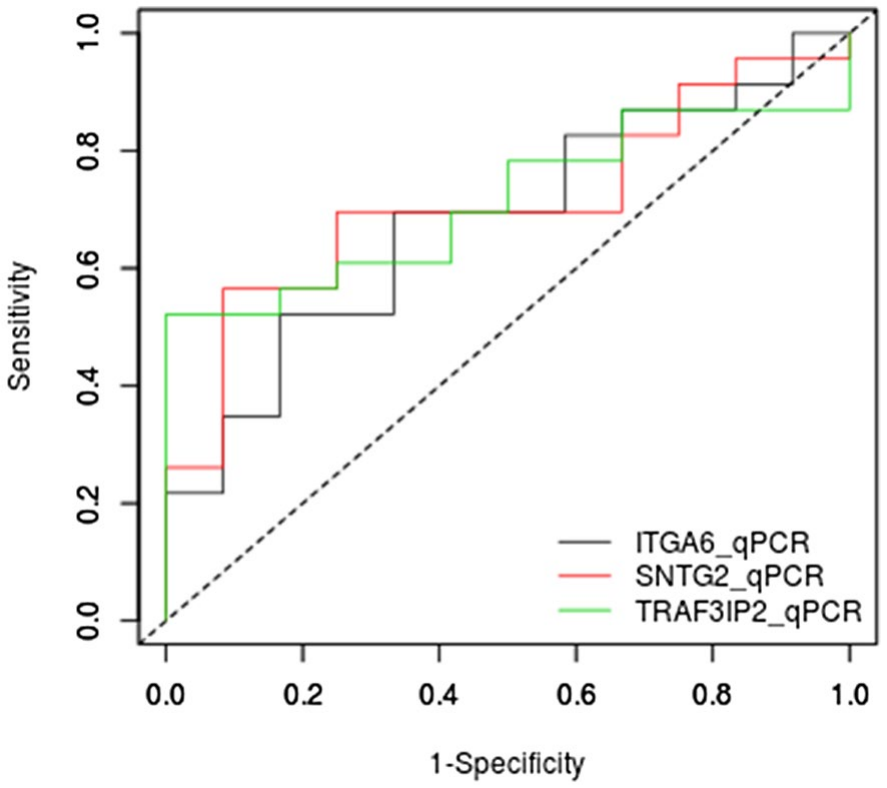
ROC curve of validated genes ITGA6, SNTG2, TRAF3IP2. Area under curve (AUC) ITGA6 = 0.68116, *p* = .05, SNTG2 = 0.71014, *p* = .01, TRAF3IP2 = 0.71377, *p* = .01)

We analyzed the sensitivity and specificity of genes for the classification of VF groups. The area under curve (AUC) for three validated genes was significant (*ITGA6* = 0.68, *p* = .05, *SNTG2* = 0.71, *p* = .01, *TRAF3IP2* = 0.71, *p* = .01). These results are shown in Figure 4.

- *ITGA6* cut‐off gene expression was 1.40, sensitivity = 70%, specificity = 67%, positive predictive value = 80%, and negative predictive value = 83%.
- *SNTG2* cut‐off gene expression was 1.86, sensitivity = 52%, specificity = 100%, positive predictive value = 52%, and negative predictive value = 100%.
- *TRAF3IP2* cut‐off gene expression was 1.40, sensitivity = 70%, specificity = 67%, positive predictive value = 80%, and negative predictive value = 83%.

## 5 DISCUSSION

This study demonstrated for the first time the overexpression of transcripts related to VFs in older women with osteoporosis. We found that overexpression of *SNTG2* strongly correlates with VF. *SNTG2* belongs to the syntrophin family, which is highly expressed in the musculoskeletal system (Moon, Choi, Heo, Kim, & Kim, 2017). Syntrophins are cytoplasmic peripheral membrane proteins that bind to workings of mechanosensitive sodium channels and the extreme carboxyl‐terminal domain of dystrophin. *SNTG2* is mainly present in the neuromuscular junction (Inoue et al., 2008). Our data suggest a potential interaction between the muscular and skeletal systems in the genesis of osteoporotic VFs.

Moreover, we also found that *TRAF3IP2, ITGA6,* and *CD248* transcripts presented high *β* ratios and upregulated expression.

Interestingly, TRAF3IP2 interacts with TNF receptor‐associated factor 3, and is a central regulator of cytokine production and type I interferons, and *TRAF3* plays a role in various autoimmune and inflammatory diseases (Boyce, 2018; Tseng et al., 2010). Furthermore, *TRAF3IP2* is highlighted in regulating RANKL‐induced osteoclast development (Xiu et al., 2014) and its expression is altered in muscle tissue through the aging process according to the GTEX database, highlighting the possible role in the pathophysiology of VFs.

Regarding to *ITGA6,* the network analysis showed that this gene is an interactor with other upregulated gene, *TNXB*, associated with focal adhesion and integrin binding. Integrins may contribute to osteoclasts/bone adhesion and increase the resorption of the bone matrix, and therefore increase fracture risk. Landowski *at al* showed that inhibition of *ITGA6* cleavage in vivo decreases osteolytic tumor activity and makes a sclerotic reaction in bone lesions (Landowski et al., 2014). The α6 integrin (*ITGA6*) is also expressed by human hematopoietic stem cell populations with plentiful laminin 511 (LN511) in healthy human bone (Qian et al., 2007). LN511 is a crucial component of the stem cell niche and maintains stem cell survival free of additional cytokines or growth factors (Graf, Heimfeld, & Torok‐Strob, 2001). Both genes were shown in the enrichment analysis as a component of the extracellular matrix, which may function by forming structure or by linking connective tissue cells.

We also found that mTOR1 signaling pathway with two genes *PNO1* and *PDK1* was significantly enriched in patients with VFs. In fact, Glantschnig*et al*. showed that through the mTOR pathway, RANKL, TNF, and M‐CSF promote the survival of osteoclasts, inhibiting the apoptosis of osteoclasts (Glantschnig, Fisher, Wesolowski, Rodan, & Reszka, 2003). It is well known, that apoptosis of bone‐related cells, including osteoclasts, osteoblasts, and osteocytes, is an important mechanism for bone mass regulation and osteoporosis. Besides, the bisphosphonates can prevent pathologic bone resorption by making osteoclast apoptosis (Hughes & Boyce, 1997) and experimental studies demonstrated that the rapamycin (an inhibitor of mTOR pathway) increased bone mass by inhibiting osteoclast differentiation (Dai et al., 2017). Furthermore, in mice with senile osteoporosis, rapamycin could restore the biological properties of aged bone marrow‐derived mesenchymal stem cells (BMMSCs) by increasing osteogenic differentiation and proliferation (Ma et al., 2018). In fact, the authors found that activation of autophagy reestablished bone loss in aged mice, proposing that autophagy mechanism plays a pivotal role in the aging of BMMSCs, and activation of autophagy could partially converse the aging (Ma et al., 2018).

The strength of our work is the comparison focused only on patients with osteoporosis (T‐score ≤−2.5), with and without VFs, since VF is the most prevalent clinical outcome in osteoporosis. In fact, the mainstream of genetic studies of bone has been showed studies estimates heritability concentrated on BMD measurements. Those differences may differ in association and magnitude of effect at different skeletal sites. It is known that about 50% of women with osteoporosis with low BMD will not develop a fracture in the future. Some studies showed that not only BMD was hereditary, but also the bone fracture risk (Oei et al., 2014). In our study, we found a result of gene expression regardless of BMD.

Furthermore, the participants of this study were a very similar group selected from the Brazilian community cohort from Sao Paulo (SPAH). Lack of selection bias enables those findings to be extrapolated to the general Brazilian population. We also excluded mild‐grade of spine deformities since some authors believe that this grade was not an actual VF, which was a limitation of previous VF study (Grados et al., 2009).

The limitations of this study include: First, the sample size was relatively small, and the data from this study should be replicated in large‐scale works and in other phenotypes with different races or from different countries. The consistency of our results might seem limited by the small number of studied individuals, but the high power of the statistical analysis value of 0.9 of the data should give cause for assurance in the accuracy of our results, which were satisfactory to find DEGs (VanIterson, 2009). Second, since we used total blood cells that do not express all bone cell proteins, some protein translation changes may have gone unnoticed during our study. However, research using whole blood mRNA has the advantage of being a less invasive method and can measure the gene expression of systemic diseases, making it more useful and safer to use in clinical practice. Also mRNA reflects the functional state of cells, and integrates responses to both genetic and epigenetic factors of gene regulation, creating it a hopeful way to explore the disease evolution (Mohr & Liew, 2007; Riedmaier & Pfaff, 2013). Other limitations of the study were very low fold changes in gene expression. We explain this effect as associated with the similar phenotype of both analyzed groups. Usage of relatively big cohorts and logistic regression enabled us to identify even very light shifts in expression. Additional validation with qPCR enabled us to select the most promising transcripts associated with the study. qPCR confirmed the direction of expression changes for most of the transcripts supporting the significance of the obtained microarray results.

In conclusion, our data enabled us to identify and validate expression changes of the transcripts *SNTG2*, *TRAF3IP2,* and *ITGA6* associated with osteoporotic VFs in elderly women, independently of BMD. These findings may serve as the basis for future longitudinal studies in the search for biomarkers of VFs, helping the early diagnosis of this comorbidity. In addition, the study of these molecules *SNTG2, TRAF3IP2, and ITGA6* can be tested as therapeutic targets in osteoporosis‐related VF.

## CONFLICT OF INTEREST

The authors (L H Jales Neto, Z Wicik, G H F Torres, L Takayama, V F Caparbo, N H M Lopes, A C Pereira, and R M R Pereira) have no financial disclosures.

## AUTHORS’ CONTRIBUTION

Study design: LHJN, NHML, ACP, and RMRP. Study conduct: LHJN, ZW, and RMRP. Data collection: LHJN, GHF, ZW, VFC, and LT. Data analysis: LHJN, ZW, and RMRP. Data interpretation: LHJN, ZW, ACP, and RMRP. Drafting manuscript: LHJN, ZW, and RMRP. Revising manuscript: LHJN, ACP, and RMRP. Approving final version of manuscript: LHJN, ZW, GHF, VFC, LT, NHML, ACP, and RMRP.

## Supporting information

Table S1

Table S2

Supplementary Material

## Data Availability

Supplemental data were included with this submission.
