# Supplementary material for: Overexpression of SNTG2, TRAF3IP2, and ITGA6 transcripts is associated with osteoporotic vertebral fracture in elderly women from community
Source: Mol Genet Genomic Med. 2020 Jun 30;8(9):e1391. doi: 10.1002/mgg3.1391 (PMC7507059; doi:10.1002/mgg3.1391)
Supplement: Supplementary file 1 — Table S1 [file MGG3-8-e1391-s001.docx]

**Supplementary table 1.** List of primer couples generated for qPCR validation of the differentially expressed genes between the older women with osteoporosis and Vertebral Fracture (VF) and osteoporosis with No Vertebral Fractures (NVF).

| Gene symbol | Tanscript | Nr | Primer sequence | product length |
| --- | --- | --- | --- | --- |
| UBXN6 | ENST00000301281.10 | FW | ACGTTCAACAAAGACCAGGA | 154 |
|  |  | RV | TCCCTTCCAGGCAGTTAATG |  |
| PNO1 | ENST00000263657 | FW | TTATTCTCGGCTTTCAGGTG | 187 |
|  |  | RV | ATCCTTGTCCGTGTCACATT |  |
| CD248 | ENST00000311330 | FW | GGTGGCTTCGAGTGTTATTG | 151 |
|  |  | RV | CCTCGTCTTCATCTTCCTCA |  |
| TNXB | X71927.1 | FW | AGATTCACTGCACCTCTCCT | 181 |
|  |  | RV | GTTCGCAATTCCAAACAGAGT |  |
| TRAF3IP2 | ENST00000340026.10 | FW | CACCCTGTGCAGAAGGTTAT | 124 |
|  |  | RV | GTGGCTGGTGATGTGGCTG |  |
| ITGA6 | ENST00000264107 | FW | GGAGATTGGAGCTTTTGTGAT | 258 |
|  |  | RV | GTTAGCAGGAACAGGAACGA |  |
| PRDX5 | ENST00000265462.8 | FW | TAGATGATTCGCTGGTGTCC | 103 |
|  |  | RV | CCATCTGGTTCCACATTCAG |  |
| RPL27 | NM_000988.5 | FW | GCAAGAAGAAGATCGCCAAG | 236 |
|  |  | RV | AAACCGCAGTTTCTGGAAGA |  |
| GAPDH | NM_002046.7 | FW | GAGTCAACGGATTTGGTCGTA | 141 |
|  |  | RV | CATGGGTGGAATCATATTGGA |  |
| RPL6 | NM_001320139.1 | FW | ATGCCTAGATATTATCCTACTGA | 204 |
|  |  | RV | CAGAGGTCCAGTCACAAGTAA |  |
| SNTG2 | ENST00000308624.9 | FW | TCTTCAAGATTTTGACTTTGAG | 217 |
|  |  | RV | GAAATCCACCGTGAAACACA |  |
