# Supplementary material for: Overexpression of SNTG2, TRAF3IP2, and ITGA6 transcripts is associated with osteoporotic vertebral fracture in elderly women from community
Source: Mol Genet Genomic Med. 2020 Jun 30;8(9):e1391. doi: 10.1002/mgg3.1391 (PMC7507059; doi:10.1002/mgg3.1391)
Supplement: Supplementary file 2 — Table S2 [file MGG3-8-e1391-s002.docx]

**Supplementary Table 2**

Results of the enrichment analysis performed on the genes differentially expressed between the older women with osteoporosis and Vertebral Fracture (VF) and osteoporosis with No Vertebral Fractures (NVF).

**Biological pathways enrichment analysis of older women with osteoporosis and Vertebral Fracture (VF) and osteoporosis with No Vertebral Fractures (NVF).**

|  | | | | | |
| --- | --- | --- | --- | --- | --- |
| **GeneSet**  **Hallmark** | **N** | **N** | **p-value** | **Adjusted-p** | **Genes** |
| mtor1 signaling | 200 | 2 | 6.02e-4 | 2.95e-2 | PNO1, PDK1 |
| **WikiPathways** | | | | | |
| Focal Adhesion | 190 | 2 | 5.19e-4 | 2.59e-2 | ITGA6, TNXB |
| **Canonical Pathways** | | | | | |
| Kegg ecm receptor interaction | 84 | 3 | 7.01e-7 | 9.31e-4 | ITGA6, GP5, TNXB |
| Kegg taste transduction | 51 | 2 | 1.04e-5 | 6.8e-3 | TAS2R60, TAS2R41 |
| Kegg hematopoietic cell lineage | 85 | 2 | 4.83e-5 | 1.07e-2 | ITGA6, GP5 |

**Gene Ontology (GO) of older women with osteoporosis and Vertebral Fracture (VF) and osteoporosis with No Vertebral Fractures (NVF).**

|  | | | | | |
| --- | --- | --- | --- | --- | --- |
| **GeneSet**  **GO biological processes** | **N** | **N** | **p-value** | **Adjusted- p** | **Genes** |
| Go sensory perception of taste | 66 | 3 | 2.64e-7 | 1.23e-2 | TAS2R60, TAS2R41, LCN1 |
| Go detection of chemical stimulus involved in sensory perceotions of taste | 45 | 2 | 7.09e-6 | 1.65e-2 | TAS2R60, TAS2R41 |
| Go cell matrix adhesion | 119 | 2 | 1.31e-4 | 2.21e-2 | ITGA6, TNXB |
| Go glycerolipid metabolic precess | 356 | 3 | 2.05e-4 | 2.64e-2 | SERINC5, TNXB, ENPP2 |
| Go regulation of phosphorus metabolic process | 1616 | 6 | 2.82e-4 | 3.15e-2 | WNK1, ITGA6, PDK1, PLCL1, TNXB, ENPP2 |
| Go cell substrate adhesion | 164 | 2 | 3.38e-4 | 3.64e-2 | ITGA6, TNXB |
| Go organelle disassembly | 185 | 2 | 4.80e-4 | 4.29e-2 | PDK1, FAM134B |
| **GO Cellular components** | | | | | |
| Go proteinaceous extracellular matrix | 355 | 4 | 1.02e-5 | 5.92e-3 | CD248, FBLN7, ITGA6, TNXB |
| Go extracellular matrix | 424 | 4 | 2.38e-5 | 6.95e-3 | CD248, FBLN7, ITGA6, TNXB |
| Go extracellular matrix component | 125 | 2 | 1.52e-4 | 1.61e-2 | ITGA6, TNXB |
| Go cell substrate junction | 398 | 3 | 3.13e-4 | 2.01e-2 | RPS29, FBLN7, ITGA6 |
| Go anchoring junction | 489 | 3 | 6.78e-4 | 3.04e-2 | RPS29, FBLN7, ITGA6 |
| **GO Molecular function** | | | | | |
| Go bitter taste receptor activity | 24 | 2 | 1.02e-6 | 1.49e-4 | TAS2R60, TAS2R41 |
| Go taste receptor activity | 30 | 2 | 2.05e-6 | 1.49e-4 | TAS2R60, TAS2R41 |
| Go phosphoric diester hydrolase activity | 90 | 2 | 5.73e-5 | 1.05e-2 | PLCL1, ENPP2 |
| Go phospholipase activity | 94 | 2 | 6.52e-5 | 1.05e-2 | PLCL1, ENPP2 |
| Go carbohydrate binding | 277 | 3 | 7.81e-5 | 1.05e-2 | CD248, LGALS14, ENPP2 |
| Go integrin binding | 105 | 2 | 9.06e-5 | 1.05e-2 | ITGA6, TNXB |
| Go lipase activity | 117 | 2 | 1.25e-4 | 1.05e-2 | PLCL1, ENPP2 |
| Go heparin binding | 157 | 2 | 2.97e-4 | 1.88e-2 | FBLN7, TNXB |
| Go cell adhesion molecule binding | 185 | 2 | 4.08e-4 | 2.47e-2 | ITGA6, TNXB |
| Go glycosaminoglycan binding | 205 | 2 | 6.47e-4 | 2.97e-2 | FBLN7, TNXB |
| Go sulfur compound binding | 234 | 2 | 9.49e-4 | 3.67e-2 | FBLN7, TNXB |
| Go receptor binding | 1474 | 5 | 1.14e-3 | 4.07e-2 | SNTG2, ITGA6, PLCL1, TNXB, TRAF3IP2 |
